# Pupillary Response as an Age-Specific Measure of Sexual Interest

**DOI:** 10.1007/s10508-015-0681-3

**Published:** 2016-02-08

**Authors:** Janice Attard-Johnson, Markus Bindemann, Caoilte Ó Ciardha

**Affiliations:** School of Psychology, University of Kent, Canterbury, CT2 7NP UK

**Keywords:** Sexual interest, Eye-tracking, Pupillary response, Sexual appeal

## Abstract

In the visual processing of sexual content, pupil dilation is an indicator of arousal that has been linked to observers’ sexual orientation. This study investigated whether this measure can be extended to determine age-specific sexual interest. In two experiments, the pupillary responses of heterosexual adults to images of males and females of different ages were related to self-reported sexual interest, sexual appeal to the stimuli, and a child molestation proclivity scale. In both experiments, the pupils of male observers dilated to photographs of women but not men, children, or neutral stimuli. These pupillary responses corresponded with observer’s self-reported sexual interests and their sexual appeal ratings of the stimuli. Female observers showed pupil dilation to photographs of men and women but not children. In women, pupillary responses also correlated poorly with sexual appeal ratings of the stimuli. These experiments provide initial evidence that eye-tracking could be used as a measure of sex-specific interest in male observers, and as an age-specific index in male and female observers.

## Introduction

The measurement of sexual arousal and observers’ sexual interests is important for psychological research and practice. For example, this is necessary to conduct research into sexual orientation causes and consequences (Mustanski, Chivers, & Bailey, 2002; Sell, 1997) and the assessment of unhealthy and inappropriate sexual desires in clinical and forensic settings (Gannon, Ward, & Polaschek, 2004; Laws & O’Donohue, 2008). Experimental psychology has contributed to this field by developing a number of assessment methods (e.g., Gress, 2005; Laws & Gress, 2004; Mokros, Dombert, Osterheider, Zappalà, & Santtila, 2010; Ó Ciardha & Gormley, 2012, 2013). Of these, viewing time, which reflects the duration for which particular content is studied, is now a widely utilized measure of interest in sexually appetitive materials (e.g., Lykins, Meana, & Strauss, 2008; Rupp & Wallen, 2007). The viewing of visual content is also accompanied by automatic changes in observers’ pupil size (Bradley, Miccoli, Escrig, & Lang, 2008), which appear to be particularly sensitive to sexual arousal (Bernick, Kling, & Borowitz, 1971). While this pupillary response was first explored 40 years ago with some elementary methods (Hess, Seltzer, & Shlien, 1965), it has received little attention since. In this study, we attempt to replicate those early findings with contemporary eye-tracking equipment to determine if it can be used to assess sexual interests. We not only wish to explore whether increased pupil size can provide an index of adults’ sexual interest in other adults but also whether this index is age-specific. This addition might be important for clinical and forensic practice.

Viewing time is a measure that is linked to a person’s interests and motivations (Henderson, 2003; Isaacowitz, 2006). In relation to sexual interest, viewing time has been used to measure interest in preferred over non-preferred figures. One way for measuring viewing time in these paradigms is to record observers’ response times while they rate the sexual appeal of pictures of men and women (Gress, 2005; Gress, Anderson, & Laws, 2013; for reviews, see Akerman & Beech, 2012; Laws & Gress, 2004; Snowden, Craig, & Gray, 2011). In these studies, longer response times for a specific stimulus type correspond to the reported sexual interest for that category (Quinsey, Ketsetzis, Earls, & Karamanoukian, 1996) and physiological measures of sexual arousal (Abel, Huffman, Warberg, & Holland, 1998). For example, heterosexual male observers tend to make slower responses when rating pictures of women than of men (Israel & Strassberg, 2009) and prepubescent children (Harris, Rice, Quinsey, & Chaplin, 1996; Quinsey et al., 1996). Female heterosexual observers also show age preferences in these viewing time paradigms (Ebsworth & Lalumière, 2012; Quinsey et al., 1996) but are inconsistent in their responses to sexually preferred and non-preferred adults (Ebsworth & Lalumière, 2012; Israel & Strassberg, 2009; Lippa, Patterson, & Marelich, 2010; Quinsey et al., 1996).

While the response time-based assessment of viewing time is an indirect measure of sexual interest, it is possible to achieve similar results more directly by tracking observers’ eye movements. During visual processing, eye gaze is directed towards scene content that matches a viewer’s personal interest (Calvo & Lang, 2004), including longer fixations on sexually preferred human figures (Fromberger et al., 2012b; Hall, Hogue, & Guo, 2011; Rupp & Wallen, 2007; for a review, see Rupp & Wallen, 2008). Heterosexual male observers, for example, view women for longer than men (Lykins et al., 2008). These viewing patterns also appear to correspond to the sexual content on display (Hall et al., 2011; Rupp & Wallen, 2007; Suschinsky, Elias, & Krupp 2007). For example, male and female observers predominantly study the faces of fully clothed persons (Hewig, Trippe, Hecht, Straube, & Miltner, 2008). However, female observers increase fixations to the body in semi-clothed stimuli (Rupp & Wallen, 2007) and male observers show a corresponding shift to pictures of nude women (Nummenmaa, Hietanen, Santtila, & Hyönä, 2012). These data therefore indicate that eye movements are sensitive to adult observers’ sexual interest in other adults.

Viewing patterns also appear to be age-specific. For example, male and female adult observers fixate on figures of their preferred age (20-year olds) more than babies and 60-year olds (Hall et al., 2011). However, whereas non-paedophilic adult males preferentially fixate on pictures of adults over children, paedophilic males show the reverse pattern (Fromberger et al., 2012a; Fromberger et al., 2013). This indicates that eye movements are not only sensitive to adult observers’ sexual interest in other adults, but can also distinguish between such interest in adults and children.

Despite these advantages, fixation behavior is an index of sexual interest that is vulnerable to top-down control. Observers could, for example, conceal their sexual interest by diverting attention to other visual content (Bindemann, Burton, Langton, Schweinberger, & Doherty, 2007). This limitation could be overcome by considering only the initial fixation to a stimulus display, which might reflect a covert and automatic orientation response to pre-attentively selected stimuli of sexual interest. In line with this reasoning, heterosexual adult males tend to direct more initial fixations at women than men (56 vs. 44 %) and young girls (57 vs. 43 %; see Fromberger et al., 2012b). However, the difference between these percentage fixations is not indicative of a sensitive measure of involuntary behavior.

In this study, we explore an alternative eye-tracking measure that might be more sensitive and not under top-down control. The pupils respond automatically to external stimulation, such as changes in lighting conditions, by increasing (dilating) or decreasing (constricting) in size. A similar pattern is also found as an arousal response to pleasant and unpleasant stimuli (Bradley et al., 2008). This dilation has been linked to the activation of the autonomic nervous system (Zuckerman, 1971) and appears to be impervious to top-down control. It has been shown, for example, that observers cannot enlarge or reduce pupil size at will in the absence of a visual stimulus (Laeng & Sulutvedt, 2014) or suppress pupil dilation (for a review, see Laeng, Sirois, & Gredebäck, 2012). These characteristics might make pupillary response an ideal measure for the assessment of sexual interest.

While this is an interesting possibility, the pupillary response to sexual arousal has received little research attention. In an early study, Hess et al. (1965) showed five hetero- and five homo-sexual males images of nude men and women while filming the observers’ eyes at a rate of two frames per second. Twenty measurements were obtained for each stimulus by manually measuring pupil diameter at each frame of the video footage. Despite this elementary approach, a clear pupillary response was found whereby all heterosexual males exhibited larger pupils to pictures of women than men. By contrast, all but one of the homosexual males showed larger pupil responses to pictures of men than women. These promising results were re-examined shortly after with the addition of female observers (Scott, Well, Wood, & Morgan, 1967). Here, observers were presented with semi-nude and clothed images of men and women. Male observers demonstrated more pupil dilation to semi-nude women than any other stimuli. Female observers did not show different pupil responses to semi-naked and clothed stimuli or male and female targets. However, a subsequent experiment also recorded a pupil dilation effect in female observers that appeared to be related to sexual interest (Hamel, 1974). In this study, female observers showed increases in pupil size that were directly related to the degree of nudity of pictures of male, but not of female, models.

Despite these promising results, there have been no attempts to replicate these findings until recently. Rieger and Savin-Williams (2012) showed hetero-, homo-, and bisexual observers sexually explicit videos, while pupillary responses were recorded with contemporary eye-tracking equipment. This study replicated the clear relationship between sexual orientation and pupil dilation that Hess et al. (1965) had found in male observers. However, similar to Scott et al. (1967), pupillary responses in heterosexual female observers were comparable when viewing footage of men and women. In a subsequent experiment, Rieger et al. (2015) extended these findings to show that pupillary responses to sexually explicit images reflect the sexual orientation of male observers, but not of heterosexual female observers, similarly to genital arousal. These findings indicate that pupillary response is a useful alternative for measuring sexual interest in male observers. In addition, the lack of specificity in heterosexual female observers converges with a broad range of assessment methods (e.g., genital arousal, self-reported sexual arousal and attraction, response time, and viewing time; Chivers, 2005; Chivers, Rieger, Latty, & Baily, 2004; Ebsworth & Lalumière, 2012; Lippa, 2006, 2007, 2012; Lippa et al., 2010; Suschinsky, Lalumière, & Chivers, 2009). This is an interesting finding because it suggests that pupillary responses to sexual content are also consistent with more established measures in the literature.

While few studies have focussed on pupil dilation as a measure of sexual interest for photographs of adults, there has been even less research on pupillary responses to persons of different ages. An early study compared these pupillary responses in incarcerated male pedophiles and non-pedophiles to images of nude women and immature girls (Atwood & Howell, 1971). This experiment revealed greater pupil dilation in 90 % of non-paedophilic observers to pictures of women, but a pupil constriction to the same pictures in 80 % of pedophiles. Conversely, images of girls produced dilation in 90 % of pedophiles and a constriction or no change in 50 % of the non-pedophilic control subjects.

Up to now, there have been no documented attempts to replicate these findings. This is surprising considering the potential applied value of such a measurement (e.g., the assessment of child sex offenders). In this exploratory study, we investigated whether pupil dilation can provide an age-specific indication of a person’s sexual interests. For this purpose, heterosexual male and female observers were presented with images of beach scenes that contained semi-clothed adults and children, while their eye movements and pupil sizes were recorded. These scenes contained only a single person or no persons in the case of a set of comparison landscape beach scenes. We expected the different person content of these scenes to draw attention depending on the sexual interests of the observers. For example, heterosexual male observers were anticipated to fixate on women more frequently than men (see Hewig et al., 2008; Lykins et al., 2008; Rupp & Wallen, 2007). Of particular interest here was whether these observers would also show an increase in *pupil size* to images of sexually preferred adults in comparison with sexually non-preferred adults and children.

As a secondary aim, we also sought to examine how pupillary responses to people of sexual interest are affected by image luminance. The pupils constrict in response to light (i.e., increased luminance) to protect the cells of the retina (Bergamin & Kardon, 2003; Ellis, 1981). If this differentially affects the stimulus categories in the current study, then this could influence the measurement of pupil responses as an index of sexual interest. In turn, it is possible that the pupillary response to sexual content is clearer when luminance is controlled across different stimulus categories. To explore this possibility, the original photographs of the beach scenes were compared with alternative versions, in which the mean luminance was equated across the different stimulus categories. This manipulation can decrease image quality by reducing light–dark contrasts. A third version of these scenes was therefore also included, in which image quality of the original photographs was enhanced with graphics software.

## Experiment 1

### Method

#### Participants

A total of 44 students (22 male and 22 female) from the School of Psychology at the University of Kent participated in this study in return for a small payment or course credits. Participants completed the Kinsey scale for the assessment of sexual orientation as part of a pre-screen on our online recruitment system. This is a seven-point scale in which a score of “0” represents complete heterosexuality and “6” complete homosexuality. Only participants who reported to be completely heterosexual (i.e., reporting “0” on the Kinsey scale) were invited to take part (Kinsey, Pomeroy, & Martin, 1948; Kinsey, Pomeroy, Martin, & Gebhard, 1953). The mean age of participants was 21.8 years (*SD* = 4.2; range 18–35 years). All reported normal or corrected-to-normal vision.

#### Materials

**Fig. 1:**
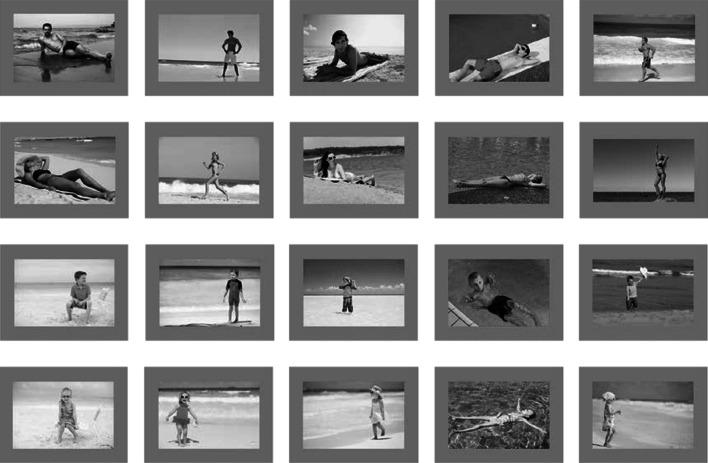
The stimuli of the original quality condition in Experiment 1

The stimuli consisted of natural beach scenes portraying men, women, and children (5 scenes for each of these four categories). To determine the approximate age of these categories, ten observers (5 males, 5 females) estimated the age of the people in the scenes in a pilot study. This revealed a mean age of 26.4 years (*SD* = 2.1) for men, 22.8 years (*SD* = 2.6) for women, 5.7 years (*SD* = 1.1) for boys, and 4.7 years (*SD* = 1.4) for girls. The age of the children therefore corresponds to stage 1 (prepubescent) of the Tanner stages of sexual development (see Tanner, 1978). Additionally, a set of control beach scenes without any person content (5 scenes) was included, resulting in a total of 25 scenes. People were portrayed in swim or leisure wear. All stimuli were purchased from an internet photograph database (www.mostphotos.com) and were selected to be of similar composition and size, and to depict the persons in similar poses and with a comparable level of clothing (see Fig. 1). To confirm that these targets were of similar size, their percentage occupancy area in the scenes was calculated. This showed that all person categories occupied a similar amount of space in our scenes (mean = 7.1 %, *SD* = 3.4, range across person categories = 6.6–7.7 %; one-factor ANOVA: *F*(3, 19) = 0.14, *p* = 0.94).

**Fig. 2:**
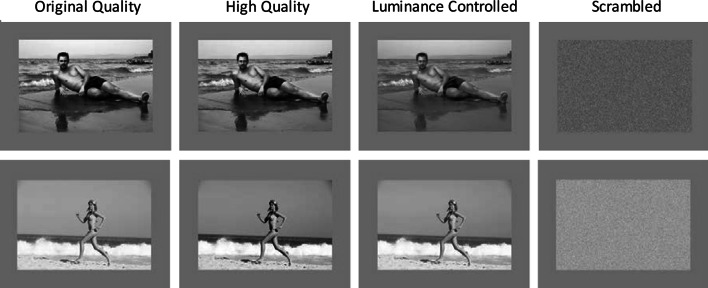
Example stimuli of the original quality, high quality, and the luminance-controlled image conditions in Experiment 1 and the scrambled images in Experiment 2

In addition, three versions were created of each scene that were identical in all aspects except for image quality. This resulted in a total of 75 scene images. In the original quality condition, the image quality of the downloaded photographs was retained. In the high-quality version, the images were processed by applying the “Auto Levels,” “Auto Contrast,” and “Auto Color” functions in Adobe Photoshop CS3 to artificially enhance the original photographs. Finally, to create a luminance-controlled version of the stimuli, the photographs were divided into groups of five (one of each category) based on similar luminance values and standard deviation. A mean luminance value and standard deviation were calculated for each of the five groups. Each photo within a group was then re-adjusted to obtain the mean luminance and standard deviation that matched the group value. Therefore, at least one image from each category (men, women, boys, girls, no-person landscapes) had precisely matched luminance values. This particular group-based approach was adopted to avoid the extreme deviation from the natural luminance values of individual scenes. This can occur when a single mean luminance value is derived for large stimulus sets, which can result in some highly distorted and unnatural looking images. Table 1 shows the overall mean luminance values and standard deviation for the different image categories for all scenes. Example stimuli are shown in Fig. 2.

**Table 1:** Mean luminance, standard deviation, and the minimum and maximum luminance values of images within a stimulus category for the original, high-quality, and luminance-controlled images for all scene conditions

	Mean	*SD*	Max	Min
Original quality				
Men	166	25	190	125
Women	160	29	200	125
Boys	169	42	218	111
Girls	190	35	224	133
No-person	165	28	190	127
High quality				
Men	167	23	186	131
Women	163	20	182	130
Boys	171	41	221	123
Girls	184	38	211	122
No-person	152	16	180	143
Luminance controlled				
Men	162	18	194	152
Women	162	18	194	152
Boys	162	18	194	152
Girls	162	18	194	152
No-person	162	18	194	152

Two questionnaires were also included in the experiment. The first was a general information scale relating to sexual interest and instructed participants to select one or more of five applicable statements (“no sexual interest in adults,” “strong sexual interest in female adults,” “some sexual interest in female adults,” “some sexual interest in male adults,” “strong sexual interest in male adults”). This was included to confirm the sexual interests that participants reported in the pre-screen. In addition, all participants completed the Interest in Child Molestation Scale to ensure that they were solely sexually interested in adults (Gannon & O’Connor, 2011). This scale consists of five short scenarios that describe incidents of child molestation. In response to these scenarios, participants have to rate their arousal, enjoyment, and behavioral propensity to child sex abuse on 7-point Likert scales. This scale has high test–retest reliability (*r* = .94) and its sexual arousal subscale correlates with the Implicit Association Test, which provides an indirect measure of child sexualization associations (see Gannon & O’Connor, 2011).

#### Eye-Tracking

The stimuli were displayed using SR-Research Experiment Builder software (version 1.1.0) on a 21″ color monitor, with a screen resolution of 1024 × 768 pixels. Eye movements were tracked using an SR-Research Eyelink II head-mounted eye-tracking system. The Eyelink II was running at a 500 Hz sampling rate, a spatial resolution of <0.01° of visual angle, a gaze position accuracy of <0.5°, and a pupil size resolution of 0.1 % of diameter. The Eyelink II system works by measuring corneal reflection and dark pupil with a video-based infrared-camera eye tracker, which computes the number of camera pixels that are occluded by participants’ pupils. In this system, the diameter of the pupil is recorded as an integer that ranges from 400 to 16,000 units. The device incorporates eye and head tracking that automatically compensates for minor head movements. During the recording of eye movements, participants are instructed to remain seated still but further immobilization (e.g., a chinrest) is not required. This eye-tracking system is compatible with most glasses and contact lenses.

#### Procedure

Participants were invited to take part in an experiment on sexual interest and informed that they would be viewing images of males and females of different ages while their eye movements were being recorded. Participants were kept naïve to the full purpose of the experiment until the end. To fully understand observers’ natural interests in these scenes, a free-viewing paradigm was used so as not to constrain spontaneous eye movement patterns. Thus, participants were instructed simply to “view the scenes as you naturally would” (for similar approaches, see Bindemann, Scheepers, & Burton, 2009; Fromberger et al., 2012a, b, 2013; Hall et al., 2011; Hewig et al., 2008; Lykins et al., 2008; Nummenmaa et al., 2012).

Participants were seated in a quiet and windowless room with consistent artificial lighting and positioned approximately 60 cm from the display monitor. The participants’ left eye was tracked and calibrated using the standard Eyelink procedure. To calibrate the eye tracker, observers fixated an initial series of nine target points on the display monitor. Their accuracy was then validated against a second series of nine fixation targets. Calibration was repeated if poor measurement accuracy was indicated. In the experiment, each trial began with a central fixation dot, which allowed for drift correction. This was followed by a gray screen display for 1000 ms, and then the stimulus display for 5000 ms, followed by another gray screen for 1000 ms. This display duration is similar to other studies with static images (e.g., Fromberger et al., 2012a, b, 2013; Hewig et al., 2008; Nummenmaa et al., 2012) and allows for approximately 15 fixations (based on an average fixation duration lasting 200–300 ms, see Rayner, 1998), which is sufficient time to scan the entire scene.

Each participant viewed all 75 stimuli. These were presented in a randomized order that was uniquely generated for each participant by the EyeLink software. Short breaks were inserted every 25 trials, after which the calibration procedure was repeated. On completion of the eye-tracking task, participants answered the general information scale relating to their sexual interests and the Interest in Child Molestation Proclivity scale (see Gannon & O’Connor, 2011).

### Results

#### Confirmation of Sexual Interests

To ensure that participants were not sexually interested in children, responses on the Interest in Child Molestation Scale were analyzed first. An overall interest score was calculated for each participant by combining responses across all subscales (i.e., arousal, enjoyment, behavioral propensity) (for similar analysis, see Gannon & O’Connor, 2011). This produced a total score where a minimum of 15 (low sexual interest in children) and a maximum score of 105 (high sexual interest in children) are possible. The results here converge with those obtained in previous studies with a sample of non-offending community males (Gannon & O’Connor, 2011), such that male observers scored a mean of 18.1 (mode = 15, *SD* = 5.6, min = 15, max = 30) and 16.8 for female observers (mode = 15, *SD* = 5.6, min = 15, max = 41). However, an established cut-off point for this scale does not exist. We adopted a simple metric by considering only individuals with scores on the lowest third of the scale (i.e., with scores between 15 and 45). All participants fell within this range.

Sexual orientation was confirmed with the general information scale that was administered following the eye-tracking task (see “Materials” section). In the 22 male observers, 19 reported “strong sexual interest in women” and three selected “some sexual interest in women.” Among the 22 females, 12 selected “strong sexual interest in males” and 10 selected “some sexual interest in males.” Participants reported no other sexual interests in this questionnaire.

#### Data Analysis

For the analysis of the eye-tracking data, all eye movements were pre-processed by merging fixations of less than 80 ms with the preceding or following fixation if it fell within half a degree of visual angle (for similar approaches, see e.g., Attard & Bindemann, 2013; Bindemann et al., 2009; Bindemann, Scheepers, Ferguson, & Burton, 2010). In addition, any fixations that fell outside the dimensions of the display monitor or that were obscured by blinking were excluded. To analyze attention to specific areas within the visual scenes, each image was then coded to define three regions of interest (ROIs), which comprised the head and body of the persons and the scene background. The mean percentage of fixations that fell on these ROIs was then calculated across observer groups (males, females) and stimulus categories (men, women, boys, girls).

For the measure of main interest, observers’ pupillary responses were computed by taking the mean pupil diameter at each fixation, averaged across the duration of a stimulus display. These values were then used to compute an overall mean, across all stimuli, for each participant. The percentage difference (i.e., an increase or decrease) in pupil diameter for each stimulus category (men, women, boys, girls, no-person scenes) from the overall mean was then computed, using the formula: (mean pupil diameter for category × 100)/overall pupil mean. Accordingly, a score of 100 % indicates that the pupillary response to a stimulus category does not differ from the overall mean. Scores higher or lower than this value indicate comparatively larger or smaller pupil sizes (for similar approaches, see Dabbs, 1997; Laeng & Falkenberg, 2007). To simplify the expression of these patterns, these scores were then deducted from 100 so that no change in pupil size is indicated by zero and positive or negative scores reflect relatively larger (dilation) or smaller (constriction) pupil sizes in response to a stimulus category.

#### Viewing Behavior

**Fig. 3:**
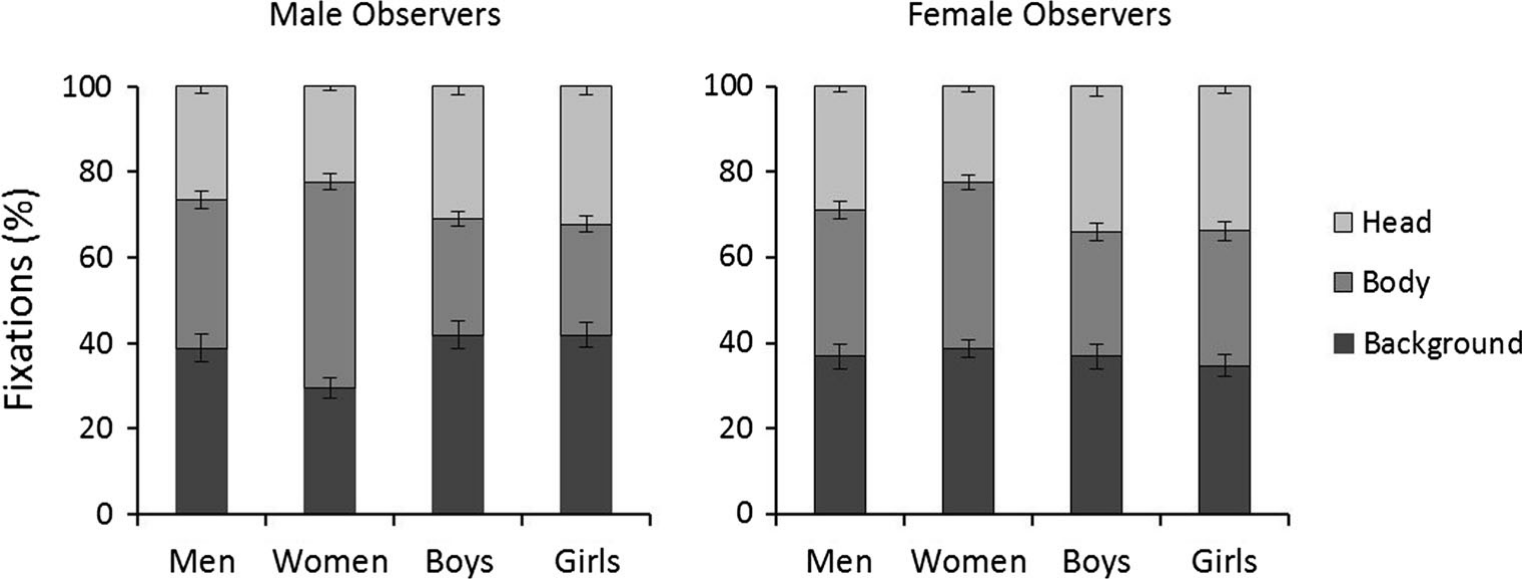
Mean percentage fixations to the head and body of the target persons and the scene background for male and female observers in Experiment 1. *Lines* represent standard errors of the means

We first examined the viewing patterns that the persons in the scenes elicited in male and female observers. To examine this, the percentage fixations to the ROIs were calculated for all stimulus categories (see Fig. 3). Overall, 63 % of fixations fell on the figures in the scenes (range 58 to 71 % across conditions), which indicates that the person content of the scenes was of most interest. A 4 (category: men, women, boys, girls) × 3 (ROI: head, body, background) × 2 (observer sex: male, female) mixed-factor ANOVA revealed a three-way interaction, *F*(6, 252) = 8.01, *p* < 0.001, partial *η*^2^ = 0.16. To explore this interaction, two separate 4 (category: men, women, boys, girls) × 3 (ROI: head, body, background) within-subjects ANOVAs were performed for male and female observers.

For male observers, this analysis showed no main effect of category, *F*(3, 63) = 0.32, *p* = 0.81, partial *η*^2^ = 0.02, but revealed a main effect of ROI, *F*(2, 42) = 4.54, *p* < 0.05, partial *η*^2^ = 0.18, and an interaction between both factors, *F*(6, 126) = 34.22, *p* < 0.001, partial *η*^2^ = 0.62. To explore this interaction, Bonferroni-adjusted pairwise comparisons of the stimulus categories were conducted for each ROI. These comparisons show that more fixations were directed at the background of scenes containing boys, girls, and men (39 to 42 %) than scenes depicting women (30 %), all *ps* < 0.01. In addition, boys (31 %) and girls (32 %) received more fixations to the head than men (27 %) and women (22 %), all *ps* < 0.01, and men’s heads were also fixated more frequently than those of women, *p* < 0.01. By contrast, male observers directed more fixations to the bodies (48 %) of women and men (34 %) than those of boys (27 %) and girls (26 %), all *ps* < 0.001, and more at women’s bodies than those of men, *p* < 0.001. None of the other comparisons reached significance, all *ps* ≥ 0.10.

The equivalent analysis for female observers showed no main effect of category, *F*(3, 63) = 0.16, *p* = 0.92, partial *η*^2^ = 0.008, but a main effect of ROI, *F*(2, 42) = 2.58, *p* < 0.001, partial *η*^2^ = 0.11, and an interaction between factors, *F*(6, 126) = 8.45, *p* < 0.001, partial *η*^2^ = 0.29. Bonferroni-adjusted pairwise comparisons of the stimulus categories show that more fixations landed on the head region of boys and girls (both 34 %) than women (22 %) and men (29 %), all *ps* < 0.001, and on the heads of men than women, *p* < 0.001. By contrast, more fixations landed on women’s bodies (40 %) compared to boys (29 %) and girls (31 %), both *ps* < 0.01. No other comparisons reached significance, all *ps* ≥ 0.08.

Overall, this pattern suggests a clear interest, whereby heterosexual males and females fixate men and women more frequently than children, but are particular biased towards the bodies of adult female targets.

#### Pupillary Responses

**Fig. 4:**
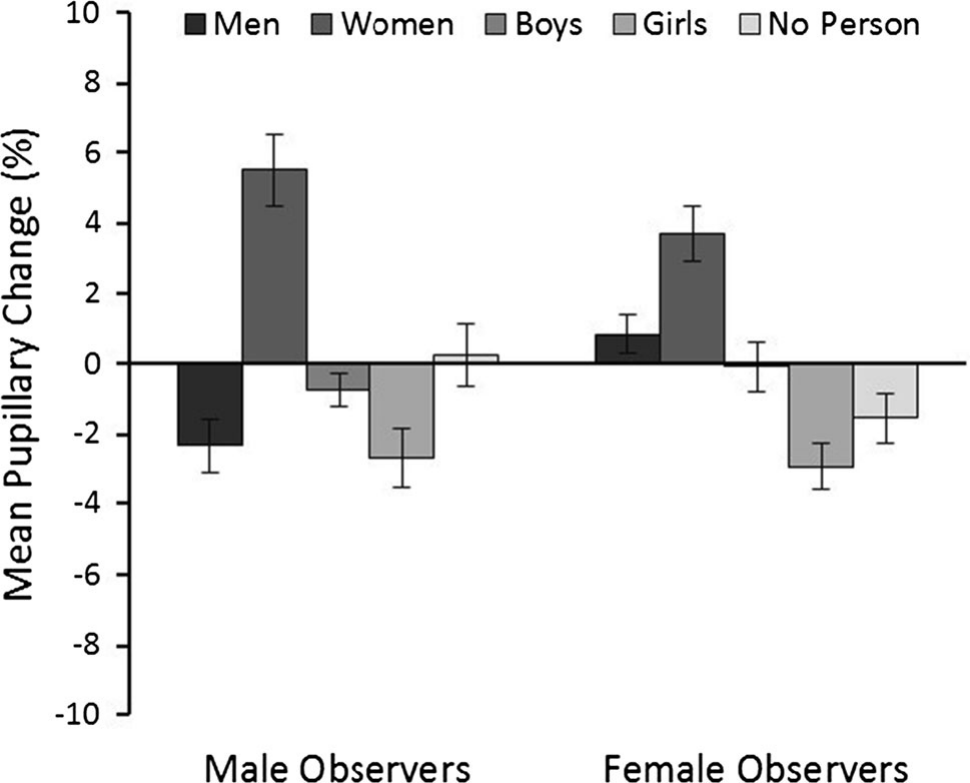
Percentage pupillary change for all stimulus categories for male and female observers in Experiment 1. *Lines* represent standard errors of the means

The measure of main interest is pupillary response, which was analyzed in two ways. In the first analysis, pupillary responses were compared for male and female observers across the stimulus categories and image conditions. These data are illustrated in Fig. 4. A 3 (image quality: original, high, luminance-controlled) × 5 (category: men, women, boys, girls, no-person) × 2 (observer sex: male, female) mixed-factor ANOVA revealed a main effect of category, *F*(4, 168) = 20.35, *p* < 0.001, partial *η*^2^ = 0.33, but not of quality, *F*(2, 84) = 1.75, *p* = 0.18, partial *η*^2^ = 0.04, or observer sex, *F*(1, 42) = 1.00, *p* = 0.32, partial *η*^2^ = 0.02. However, an interaction between image quality and observer sex was found, *F*(2, 84) = 3.36, *p* < 0.05, partial *η*^2^ = 0.07. Bonferroni-adjusted pairwise comparisons revealed only that female observers exhibited larger pupils than male observers during the viewing of luminance-controlled scenes, *p* < 0.05. No other differences were significant, all *p*s ≥ 0.09. An interaction between image quality and category was also found, *F*(8, 336) = 2.17, *p* < 0.05, partial *η*^2^ = 0.05, as the no-person beach scenes elicited smaller pupils in the luminance-controlled than the high quality, *p* < 0.01, and original quality conditions, *p* < 0.05. No other differences between any of the person content scenes were found, all *p*s ≥ 0.16. Therefore, image quality was not analyzed further.

An interaction between category and observer sex was also present, *F*(4, 168) = 2.73, *p* < 0.05, partial *η*^2^ = 0.06. Bonferroni-adjusted pairwise comparisons revealed smaller pupils in male than female observers during the viewing of men, *p* < 0.01. Furthermore, in male observers, women elicited larger pupil sizes than men, boys, girls, and no-person scenes, all *p*s ≤ 0.001. For female observers, women elicited larger pupil sizes than boys, girls, and no-person scenes, all *p*s ≤ 0.05, but not men, *p* = 0.26. In addition, pupil responses were larger for scenes depicting boys than girls, *p* < 0.05. No other differences were observed, all *ps* ≥ 0.06, and an interaction between the three factors was not found, *F*(8, 336) = 1.10, *p* = 0.36, partial *η*^2^ = 0.03. Overall, these results therefore reveal a dilation response in male observers that appears to be consistent with self-reported sex and age preferences. Female observers’ responses are also consistent with their age preferences, but do not correspond with their reported sexual interest in adult men.

In the second analysis, this pattern is confirmed when pupillary responses are compared via one-sample *t*-tests (with *alpha* corrected at *p* < 0.01 for multiple comparisons) with a baseline that reflects the mean pupil diameter across all stimuli (see “Data Analysis” section). This analysis shows that the pupils of male observers were larger than baseline during the viewing of women, *t*(21) = 5.43, *p* < 0.001, *d* = 2.37, and smaller during the viewing of men, *t*(21) = −3.02, *p* = 0.006, *d* = 1.32, and girls, *t*(21) = −3.1, *p* = 0.005, *d* = 1.35. In addition, pupil size was unchanged from baseline in response to boys and no-person scenes, both *t*s ≤ −1.59, *p*s ≥ 0.126, *ds* ≤ 0.69. In female observers, pictures of men*, t*(21) = 1.49, *p* = 0.15, *d* = 0.65, boys, *t*(21) = −0.12, *p* = 0.91, *d* = 0.05, and landscape beach scenes (−1.53 %), *t*(21) = −2.19, *p* = 0.04, *d* = 0.96 did not elicit a change in pupil size from baseline. The pupils were enlarged to scenes with women, *t*(21) = 4.71, *p* < 0.001, *d* = 2.06, and smaller than baseline during the viewing of girls, *t*(21) = −4.33, *p* < 0.001, *d* = 1.89.

#### Individual Differences in Pupillary Responses

We also sought to explore whether pupillary responses can be informative about the sexual interests of individual observers. For this purpose, the difference in raw pupil size for specific image comparisons (e.g., scenes with men vs. women) was calculated separately for each participant. These data show, for example, that all of the male observers (22/22) recorded larger pupil sizes during the viewing of women than men, and 91 % (20/22) of male observers displayed larger pupils in response to women than girls. In addition, only 22 % (5/22) of these participants showed a greater pupillary response to men than boys. With regard to female observers, 73 % (16/22) showed more pupil dilation during the viewing of women than men. However, 86 % (19/22) of this participant group also exhibited larger pupils in response to women than girls, and 59 % (13/22) recorded larger pupils to men than boys.

### Discussion

The purpose of this experiment was to explore whether pupillary responses to the visual presentation of men and women can provide an indication of a person’s sexual interests. More specifically, we sought to determine whether this approach can be extended to reveal age-specific sexual interests. We first looked at fixation patterns on the person content in scenes. Male observers showed a viewing preference for women over men and children, which was characterized by a high number of fixations on women’s bodies. These results are consistent with other studies, which have shown that heterosexual male observers attend more to images of the opposite sex (Lykins, Meana, & Strauss, 2006; Lykins et al., 2008; Rupp & Wallen, 2007; Suschinsky et al., 2007) and that such preferential viewing behavior is also age-specific (Fromberger et al., 2012a, b, 2013; Hall et al., 2011). Female observers also recorded fewer fixations on the faces of women than men and children, but more on women’s bodies than those of children. Consistent with previous research, heterosexual females therefore showed age-specific viewing patterns but did not exhibit the same strong visual preferences to opposite-sex figures as men (Hall et al., 2011; Israel & Strassberg, 2009; Lykins et al., 2008; Rupp & Wallen, 2007).

The data of main interest were the pupillary responses. In heterosexual male observers, these responses were consistent with their reported sexual interests. Thus, pictures of women elicited a clear pupillary dilation that was not present during the viewing of men and children. In female observers, pupil dilation was also greatest when pictures of women were viewed. In these participants, pupillary recordings therefore do not correspond to their self-reported sexual orientation. However, these responses still appeared to be age-specific as the pupils remained unchanged or constricted during the viewing of children.

These results converge with a recent study that has shown a similar pattern of pupillary responses for heterosexual adult males and females (Rieger & Savin-Williams, 2012). Experiment 1 extends these findings by demonstrating that such pupillary responses are also age-specific. A question that arises, however, is whether these dilation effects could be attributed to a low-level factor such as luminance. To explore this possibility, we also compared scene photographs in which contrast and color were enhanced with a set in which luminance and contrast were equated. The results for these stimulus categories were highly comparable, which suggests that pupillary responses for the different person categories cannot be explained by general variation in luminance.

There is, however, a problem with the luminance adjustment that was employed in Experiment 1. While this manipulation was used to equate luminance across scenes, it does not control other low-level image aspects, such as color, which might also affect pupillary responses (Kohn & Clynes, 1996; Lobato-Rincón et al., 2014). Such information was not matched across stimulus categories in Experiment 1. Consequently, the possibility remains that the results might reflect such image artifacts.

A second explanation is also possible for the observed pupillary responses. While we adjusted the mean luminance of the scenes, we did not measure the sexual attractiveness of the target figures. As a result, this might have been mismatched across categories. Considering that photographs of women elicited more pupil dilation in *both* male and female observers, it is conceivable, for example, that these pictures were generally more sexually arousing than those of men. To investigate these possibilities, a second experiment was conducted.

## Experiment 2

In Experiment 2, a new condition was created, in which the pixels of the luminance-controlled images were randomized. These *scrambled* images are no longer recognizable as the original scenes but provide the same color content. If the pupillary responses in Experiment 1 reflect a low-level color artifact, then the same pattern should persist with these scrambled scenes in Experiment 2. The experiment also examined whether the pictures of men and women in Experiment 1 were matched in terms of their perceived attractiveness. For this purpose, two measures of attractiveness were employed. The first measured *general* sexual appeal and recorded how attractive observers thought the stimuli were to others (i.e., sexual appeal by “societal standards”; for similar approaches, see Lippa et al., 2010). The second measure concerned the sexual appeal that these images personally hold for the *individual* observer (see Ebsworth & Lalumière, 2012; Hewig et al., 2008). If the pupillary responses in Experiment 1 reflect sexual arousal, then personal sexual appeal ratings should correlate with pupillary responses in Experiment 2.

### Method

#### Participants

A total of 41 students (21 male) from the University of Kent participated in this study in return for a small payment or course credits. The mean age was 19.5 years (*SD* = 2.0; range 18–31 years). All participants reported to be exclusively heterosexual on the Kinsey scale (Kinsey et al., 1948, 1953), which was completed as a pre-screen on our online recruitment system. None of the participants had taken part in the first experiment. All reported normal or corrected-to-normal vision.

#### Materials

This experiment employed the same eye-tracking set-up with the luminance-controlled stimuli from Experiment 1. To assess the contribution of color *within* each of these 25 images (comprising five men, women, boys, girls, and no-person scenes) to pupillary response, the pixels in each image were randomized. The resulting images provide a “scrambled” condition in which the original image content is not discernible (see Fig. 2; for similar approaches, see Jenkins, Lavie, & Driver, 2003; VanRullen, 2006).

#### Procedure

The experiment consisted of four blocks. In the first block, participants were shown the 25 scrambled scene images. This was followed, in the second block, by the 25 unscrambled versions of these stimuli. Both blocks were free-viewing tasks. Each trial therefore consisted of a drift correction, which was followed by a gray mask for 1000 ms. The scrambled/intact scene stimuli were then presented for 5000 ms, followed by the gray mask for a further 1000 ms. In both blocks, participants were simply instructed to view these images naturally.

In the remaining blocks, the intact scenes with the men (5 images), women (5 images), and children (5 images each) from Block 2 were repeated. In Block 3, participants were asked to provide personal sexual attractiveness ratings for these people (i.e., based on how sexually attractive they themselves find these images) using a Likert scale ranging from 1 (“not at all sexually appealing to me”) to 7 (“extremely sexually appealing to me”). In Block 4, participants were then asked to evaluate the people in the scenes based on their sexual attractiveness by societal standards using the same scale (for similar methods, see, e.g., Lippa et al., 2010). For all four tasks, the stimulus sequence in each block was generated randomly by the display software for each participant. As in Experiment 1, participants completed the same general information scale and the Interest in Child Molestation proclivity scale on completion of the eye-tracking tasks.

### Results

#### Confirmation of Sexual Interests

Once again, the responses on the Interest in Child Molestation Scale were analyzed first. One of the male participants produced a score of 52. This is the only score that falls above the lowest third (i.e., 45) of the Child Molestation Scale in Experiment 1 and 2. It also exceeds the mean score (41.4) of pedophiles that have self-reported sexual acts with children (Mitchell & Galupo, 2015). This individual was therefore excluded from further analysis. For the remaining participants, means of 20.8 (mode = 15, *SD* = 6.2, min = 15, max = 34) and 16.3 (mode = 15, *SD* = 2.4, min = 15, max = 23) were obtained for male and female observers, respectively.

To confirm that participants showed a sexual interest towards the opposite sex, their responses on the sexual interests’ questionnaire were also analyzed. Nineteen of the 20 males reported “strong sexual interest in women” and one reported “some sexual interest in women.” For the females, 14 of 20 reported “strong sexual interest in males,” while the remaining six participants reported “some sexual interest in males.” Participants reported no other sexual interests in this questionnaire.

#### Data Analysis

The eye-tracking data were processed as in Experiment 1. Note that pupillary responses are reported for both free-viewing tasks (Block 1 and 2) but not for the two ratings tasks. In the latter tasks, 5.9 (*SD* = 3.7) and 6.5 (*SD* = 4.3) fixations were recorded on average per trial but the mean number of fixations varied greatly across observers (from 1 to 38). Consequently, these tasks did not provide reliable eye movement data for analysis. The eye fixations for the free-viewing task with the intact scenes (Block 2) were also analyzed and revealed a similar pattern to Experiment 1. For brevity, this analysis is not reported here but is available on request. These data are not meaningful for the scrambled scene images in Block 1 and are therefore also omitted.

#### Pupillary Responses

**Fig. 5:**
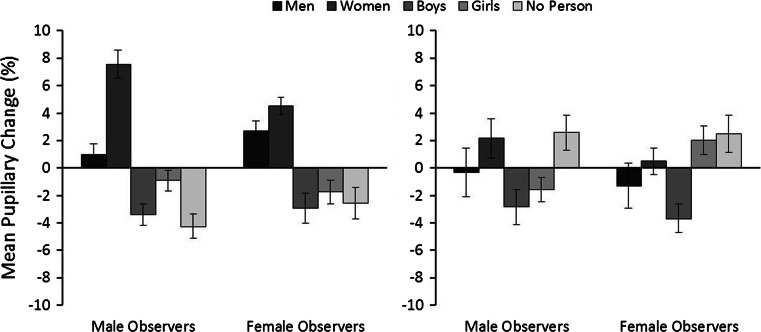
Percentage pupillary change for all stimulus categories for male and female observers in Experiment 2 for intact scenes (*left graph*) and scrambled scenes (*right graph*). *Lines* represent standard errors of the means

The data of main interest were the pupillary responses. As in Experiment 1, the mean percentage change in pupil size was calculated for male and female observers for the person categories (see Fig. 5) and was analyzed in two ways. First, a 5 (category: men, women, boys, girls, no-person) × 2 (observer sex: male, female) mixed-factor ANOVA showed a main effect of category, *F*(4, 152) = 32.16, *p* < 0.001, partial *η*^2^ = 0.46. Post hoc analysis revealed overall larger pupils during the viewing of women compared to all other categories, all *p*s ≤ 0.001, and larger pupils to men than boys, girls and no-person scenes, all *p*s ≤ 0.01. No other differences were found, all *p*s ≥ 0.34. A main effect of observer sex, *F*(1, 38) = 0.05, *p* = 0.82, partial *η*^2^ = 0.001, and an interaction between factors, *F*(4, 152) = 2.01, *p* = 0.96, partial *η*^2^ = 0.05, was not found.

For completeness, these responses were also analyzed with one-sample *t*-tests (with *alpha* corrected at *p* < 0.01 for multiple comparisons), by comparing the change in pupil size for each stimulus category with a baseline of zero (see “Data Analysis” section). For male observers, this analysis revealed pupil dilation during the viewing of women, *t*(19) = 7.58, *p* < 0.001, *d* = 3.48, and pupil constriction during the viewing of boys, *t*(19) = −4.40, *p* < 0.001, *d* = 2.02 and no-person scenes, *t*(19) = −4.62, *p* < 0.001, *d* = 2.12. A change in pupil size was not detected in response to images of men, *t*(19) = 1.26, *p* = 0.22, *d* = 0.58 and girls, *t*(19) = −1.23, *p* = 0.24, *d* = 0.56.

In female observers, dilation was also observed in response to pictures of women, *t*(19) = 7.25, *p* < 0.001, *d* = 3.33. However, in this case, dilation was also found for pictures of men, *t*(19) = 3.30, *p* = 0.004, *d* = 1.51. In contrast, the pupils appeared to be smaller than baseline during the viewing of boys, *t*(19) = −2.65, *p* = 0.02, *d* = 1.22, girls, *t*(19) = −2.05, *p* = 0.05, *d* = 0.94, and the no-person scenes, *t*(19) = −2.25, *p* = 0.04, *d* = 1.03, but these changes were not significantly below zero (with *alpha* corrected at *p* < 0.01 for multiple comparisons).

In summary, this analysis shows that male observers’ pupils dilate in response to pictures of women but not men or children. Female observers show a dilation response to both men and women, but not to children. These results therefore replicate the sex-specific effect in male observers and the age-specific pattern that was observed in male and female observers in Experiment 1.

#### Individual Differences in Pupillary Responses

As in Experiment 1, we also performed a simple analysis of individual performance, based on the differences between stimulus categories in raw pupil diameter during the free-viewing task (Block 2). These data show that 80 % (16/20) of the male participants displayed larger pupils when viewing women than men, 95 % (19/20) displayed larger pupils to women than girls, and 85 % (17/20) displayed larger pupils to men than boys. Of the female observers, 65 % (13/20) recorded larger pupils to women than men, 90 % (18/20) displayed larger pupils to women than girls, and 90 % (18/20) displayed larger pupils to men than boys.

#### Personal Sexual Appeal Ratings

In the next step of the analysis, we explored the extent to which personal sexual appeal judgements of the persons in the scenes relate to pupil responses in the free-viewing task. For this purpose, the mean sexual appeal ratings for each of the person categories were analyzed first. A 4 (category: men, women, boys, girls) × 2 (observer sex: male and female) mixed-factor ANOVA of these data did not show a main effect of observer sex, *F*(1, 38) = 0.02, *p* = 0.88, partial *η*^2^ = 0.00, but revealed a main effect of category, *F*(3, 114) = 83.26, *p* < 0.001, partial *η*^2^ = 0.69, and an interaction between factors, *F*(3, 114) = 87.53, *p* < 0.001, partial *η*^2^ = 0.70. Bonferroni-corrected post hoc comparisons showed that male observers rated women as more sexually appealing (*M* = 5.4, *SD* = 0.9) than men (*M* = 1.6, *SD* = 0.8), boys (*M* = 1.2, *SD* = 0.8), and girls (*M* = 1.2, *SD* = 0.7), all *p*s < 0.001. In contrast, female observers rated men as more sexually appealing (*M* = 4.3, *SD* = 1.40) than women (*M* = 2.1, *SD* = 1.2), boys (*M* = 1.3, *SD* = 0.9), and girls (*M* = 1.5, *SD* = 1.3), all *p*s < 0.001. No other differences were found. Overall, these sexual appeal ratings therefore converge clearly with observer’s self-reported sexual interest in adults of the opposite sex.

We next performed a correlation between the mean pupillary change (%) in the free-viewing task (Block 2) and the sexual appeal ratings.^1^ This analysis combined the person categories (men, women, boys, girls) but was performed separately for male and female observers. The distribution of observers’ sexual appeal ratings was skewed. Therefore, non-parametric Spearman’s correlations are reported. For male observers, a strong positive correlation between pupil change and sexual appeal ratings was found, *r*_*s*_(78) = 0.64, *p* < 0.001. This correlation also persisted when only the adult targets (men and women) were considered, *r*_*s*_(38) = 0.58, *p* < 0.001, which suggests that it reflects observers’ sexual interests in specific adults. For female observers, the correlation across all person categories (men, women, boys, girls) was weaker, *r*_*s*_(78) = 0.28, *p* < 0.01, and was not reliable when the child categories were excluded from analysis, *r*_*s*_(38) = −0.22, *p* = 0.17. Overall, these data therefore suggest that pupillary responses provide a good index of sexual interest in male, but not female, observers.

#### General Sexual Attractiveness Ratings

In Block 4, the subjects were asked to objectively rate the persons in the scenes on their sexual attractiveness based on how they thought the general population would respond. The mean ratings were analyzed with a 4 (category: men, women, boys, girls) by 2 (observer sex: male and female) ANOVA. This analysis did not show a main effect of observer sex, *F*(1, 38) = 0.45, *p* = 0.51, partial *η*^2^ = 0.01, but a main effect of category, *F*(3, 114) = 331.15, *p* < 0.001, partial *η*^2^ = .90, and an interaction between factors, *F*(3, 114) = 2.96, *p* < 0.035, partial *η*^2^ = 0.07. Bonferroni-corrected post hoc comparisons revealed that male observers rated the women in scenes (*M* = 6.0, *SD* = 0.6) higher on sexual attractiveness than men (*M* = 4.8, *SD* = 1.02), *p* < 0.001. Both adult categories were also rated higher than boys (*M* = 1.4, *SD* = 0.9) and girls (*M* = 1.4, *SD* = 0.9), all *p*s < 0.001. Female observers rated men (*M* = 5.6, *SD* = 1.0) and women (*M* = 5.7, *SD* = 1.1) more similarly (*p* = 1.00), and more sexually attractive than boys (*M* = 1.4, *SD* = 1.0) and girls (*M* = 1.5, *SD* = 1.2), both *p*s < 0.001. No other differences were observed.

A non-parametric Spearman’s correlational analysis between these ratings and observers’ pupillary responses (% change), which combined the data from all person categories (men, women, boys, girls), revealed a correlation for male and female observers, *r*_*s*_(78) = 0.62, *p* < 0.001 and *r*_*s*_(78) = 0.55, *p* < 0.001, respectively. Similar to the previous analysis, we performed a second correlation for which the data for child targets were excluded. This correlation was not significant in male, *r*_*s*_(38) = 0.29, *p* = 0.08, or female observers, *r*_*s*_(38) = 0.07, *p* = 0.67.

#### Scrambled Scenes

The pupillary responses to scrambled scenes were analyzed next. As in the analysis of intact scenes, the mean pupillary responses for each category (men, women, boys, girls, no-person scenes) were transformed to measure mean percentage change (see Fig. 5). A 5 (category: men, women, boys, girls, no-person) × 2 (observer sex: male, female) mixed-factor ANOVA did not show a main effect of observer sex, *F*(1, 38) = 0.00, *p* = 1.00, partial *η*^2^ = 0.001, or an interaction between factors, *F*(4, 152) = 0.97, *p* = 0.43, partial *η*^2^ = 0.03, but revealed a main effect of category, *F*(4, 152) = 4.34, *p* < 0.01, partial *η*^2^ = 0.10. Post hoc Bonferroni comparisons showed that observers’ pupils were smaller while viewing scrambled images of boys than those of women, *p* < 0.01, and no-person scenes, *p* < 0.01. No other differences between categories were found, all *ps* ≥ 0.20.

Once again, these responses were also analyzed via a series of one-sample *t*-tests (with alpha corrected at *p* < 0.01) to compare the change in pupil size to a baseline of zero (see “Data Analysis” section). This analysis showed no change in pupil size across categories in male observers, all *t*s ≤ 2.23, *p*s ≥ 0.04, *d*s ≤ 1.02. The pupils of female observers were smaller during the viewing of scrambled scenes of boys, *t*(19) = 3.46, *p* < 0.01, but no other differences were found, all *t*s ≤ 1.83, *p*s ≥ 0.08, *d*s ≤ 1.59. We also correlated pupil sizes for scrambled and intact scenes. This revealed no relationship between these conditions in male and female observers,* r*(98) = 0.06, *p* = 0.58 and *r*(98) = 0.04, *p* = 0.72, respectively. These results therefore indicate that pupillary responses to intact scenes do not reflect low-level image artifacts, such as color.

### Discussion

This experiment assessed further whether observers’ pupillary responses reflect their sexual interest in a seen stimulus. For this purpose, we compared pupillary responses to pictures of men and women with personal sexual appeal ratings and general attractiveness ratings (by societal standards). The pupils of male observers dilated to pictures of women but not men or children. Female observers showed pupillary dilation to pictures of women and men but not to children. This experiment therefore replicates the age-specific dilation effects in male and female observers that were shown in Experiment 1, and also the sex-specific dilation effect in males.

The personal sexual appeal ratings support the notion that these pupillary responses reflect the sexual interests of heterosexual male observers (Rieger et al., 2015; Rieger & Savin-Williams, 2012). For example, these observers rated the photographs of women as much more sexually attractive than those of men and children, and these ratings correlated strongly with pupillary responses. This was evident when data from all person categories were combined, but also when the children were omitted from the analysis. This suggests that the pupillary responses of male observers reflect the sexual interest that is triggered by the stimuli.

In line with their reported sexual orientation, heterosexual female observers rated male targets as most sexually appealing, while women and children received low ratings. These ratings diverge from their pupillary responses, which indicate dilation to pictures of men *and* women. In addition, a correlation between sexual appeal ratings and pupillary responses was found, but this did not hold when child categories were excluded from analysis. This pattern deviates from our findings with heterosexual male observers. It is interesting to note, however, that such discrepancies were also obtained for pupil dilation and subjective arousal in a recent experiment (Rieger et al., 2015) and are commonly observed in studies comparing self-reported and physiological measures of sexual arousal in heterosexual women (Rieger et al., 2015; Suschinsky & Lalumière, 2012; Suschinsky et al., 2009; for a meta-analysis, see Chivers, Seto, Lalumière, Laan, & Grimbos, 2010).

We also investigated whether the pupillary responses of male and female observers might reflect differences in the *general attractiveness* of the stimulus categories, by measuring how sexually attractive observers thought the stimuli were to others. Male observers rated children and adult males as less generally attractive than adult females. However, the difference between male and female stimuli was smaller than for the personal appeal ratings, indicating some adjustment. This difference was smaller still in female observers, who perceived men and women to be of similar general sexual attractiveness. Moreover, while the general attractiveness ratings correlated with pupillary responses, this did not hold for male or female observers when the child categories were excluded from analysis. This suggests that the general sexual attractiveness of male and female adult stimuli was not grossly mismatched in the current experiments, or that this was the key determinant of pupillary responses.

We also explored whether the pupillary pattern could arise from low-level artifacts within the scene images (Kohn & Clynes, 1969; Lobato-Rincón et al., 2014). To investigate this possibility, a control condition of scrambled images was included, which are no longer recognizable as coherent scenes but retain their color content. These scrambled scenes failed to produce pupillary dilation that corresponds with responses to the intact scenes. These findings therefore converge with the sexual appeal and attractiveness ratings to indicate that the pupillary responses in this study are driven by the person content of the scenes.

## General Discussion

The study examined whether pupillary responses to photographs of people can provide an indication of an observer’s sexual interests. We specifically sought to determine whether such responses are sensitive to the age of targets. Experiment 1 showed that pupils of heterosexual male observers dilated during the presentation of women but not during the viewing of men and children. This suggests that these pupillary responses are linked to the sexual interest of these observers (i.e., females) and are also age-specific (adults). In contrast, the pupils of heterosexual female observers dilated to images of women and men, but not to children. In these observers, pupillary responses therefore appear to be age-specific but do not correspond to self-reported gender interests.

In light of these different effects in male and female observers, a further experiment was conducted to explore more directly whether pupillary responses are linked to observers’ sexual interest. For this purpose, we recorded pupillary responses to male and female adults and children and also asked observers to rate these target persons in terms of their sexual attractiveness. Two measures were utilized for this purpose, which sought to capture the sexual attractiveness that these stimuli personally held for an observer as well as their general sexual attractiveness to others. The pupillary responses in this experiment replicated the sex- and age-specific effect in male observers and the age-specific effect in female observers. This suggests, once again, that pupillary response can provide a measure of sexual interest for male but not female observers.

These findings received further support from the ratings tasks. The relationship between personal sexual appeal ratings and pupillary responses was weak for females and driven by the age of the persons in the scenes. However, the ratings of male observers showed a clear preference for adult females and correlated well with pupillary response, which suggests that it reflects the sexual interests of the males in this study. By contrast, male and female observers perceived the general sexual attractiveness of men and women to be more comparable and these ratings did not correlate with pupillary response. Taken together, these findings suggest that pupillary responses reflect the personal sexual interests of male but not female observers, but are age-specific in both groups.

The responses of male observers to images of women converge with previous research, which has also shown an increase in pupil size to such content (Hess et al., 1965; Rieger & Savin-Williams, 2012; Rieger et al., 2015). Female observers recorded pupil dilation in response to images of men in Experiment 2 but also displayed larger pupils for images of women across both experiments. The reason for this is unclear. However, this absence of sex-specific pupillary responses for female observers is also consistent with other paradigms in this field, such as viewing time studies (Israel & Strassberg, 2009; Lippa et al., 2010), as well as self-reports and physiological arousal (Chivers et al., 2004, 2010; Steinman, Wincze, Sakheim, Barlow, & Mavissakalian, 1981; Suschinsky et al., 2009). For example, in these studies, women frequently show increased physiological arousal to images of both sexes (e.g., Chivers et al., 2004; Wincze & Qualls, 1984) and weaker correlations than men with self-reported preference and sexual arousal (Chivers et al., 2004; Schmidt, 1975). These findings indicate that women’s sexual interests are organized differently to those of men (Lippa, 2006, 2007; Suschinsky et al., 2009) and may not be as strongly linked to arousal patterns (for a review, see Chivers, 2005). The current experiments suggest that this also applies to pupillary responses.

It is noteworthy that our pupillary responses in males and females are also consistent with a small set of studies from the 1960s, which first assessed pupil dilation with an elementary video-frame analysis (Hess et al., 1965; Scott et al., 1967), and a recent study that verified these findings with contemporary eye-tracking equipment (Rieger & Savin-Williams, 2012). The current experiments extend this recent work by demonstrating that such pupillary responses are also age-specific, whereby the pupils of non-pedophilic observers dilate to pictures of adults but not children. This age-specific effect represents, in fact, the most consistent aspect of our results.

This is an important finding that raises the possibility that pupillary response could be used as a measure of deviant sexual interest in children in the assessment and rehabilitation of offending populations (Gannon et al., 2004; Laws & O’Donohue, 2008). To this point, it is notable that the lack of pupil dilation by male observers during the viewing of boys and girls is consistent with an old study that compared pedophilic and non-pedophilic males with a more elementary approach (Atwood & Howell, 1971). In that study, pupillary response appeared to provide an index of age-specific sexual interests in 77 % of individual observers. The current study also recorded larger pupillary responses to women than men in the majority of male observers (100 and 80 % of participants in Experiment 1 and 2, respectively), and to women than girls (91 and 95 % of participants in Experiment 1 and 2).

### Limitations and Directions for Future Research

This is an exploratory study with limitations. For example, we sought to increase ecological validity by using images of beach scenes, as these provide a natural setting to display semi-nude people (i.e., wearing only beachwear) to enhance sexual arousal. However, this approach also resulted in variation of the person content in terms of body posture, facial expression, eye gaze of the targets, and so forth. This could have affected eye fixations around the scenes and pupillary responses (Birmingham, Bischof, & Kingstone, 2008). This could be addressed in future studies by using more controlled stimuli. As an alternative, such experiments could compare pupillary responses of hetero-, homo-, and bisexual male observers. If pupillary response provides a robust measure of sexual interest, rather than reflecting other factors within natural scenes, then this should reflect the specific sexual interests of these different observer groups.

A small set of studies have shown that the pupils appear to be resistant to top-down control, such that observers cannot willingly increase or decrease their pupil size (Laeng et al., 2012; Laeng & Sulutvedt, 2014). However, the possibility still exists that observers can manipulate such responses voluntarily by avoiding person content in the visual field (Bindemann et al., 2007), or by causing pupil constriction through focusing on high-luminance scene regions. Considering that participants in this study were naïve to the full purpose of the experiment until the end, it is unlikely that such methods were adopted to exert top-down control on pupillary responses. Nonetheless, this is clearly another important avenue for further investigation.

We have also only been able to demonstrate pupillary responses with male adult observers who are sexually interested in other adults but *not* in children. We therefore acknowledge that further work with a pedophilic population and contemporary eye-tracking equipment is required to determine fully whether pupillary responses can detect such inappropriate sexual interests. In future research, it would also be valuable to compare pupil dilation directly with other existing measures of deviant sexual interest, such as Implicit Association Tests (Babchishin, Nunes, & Herman, 2013), Stroop Tasks (Ó Ciardha & Gormley, 2012; Price & Hanson, 2007), and Choice Reaction Time tasks (Mokros et al., 2010; Wright & Adams, 1994). This may serve to strengthen the validity and assessment value of these diagnostic measures, and would also help to establish the comparative strength of a pupil dilation paradigm.

### Conclusion

This is the first study to show with contemporary eye-tracking equipment that pupillary responses provide a promising method for measuring age-specific sexual interests. We have only been able to demonstrate this with male adult observers who are sexually interested in other adults and not in children. We therefore acknowledge that further work is required to determine fully whether pupillary responses can detect pedophilic sexual interests. However, pupil dilation appears to be a highly promising method for assessing such deviant sexual interests. This measure seems to relate directly to observers’ sexual interest in other adults and genital arousal (Rieger et al., 2015). It is also an autonomic response that operates outside of conscious control (Laeng et al., 2012; Laeng & Sulutvedt, 2014). Consequently, pupil dilation might provide a more robust measure of deviant sexual interest than current measures, which are prone to social desirable responding and participant manipulation (for a review, see Kalmus & Beech, 2005). Our data also suggest that pupillary response could be a sensitive measure at an individual level. This is an important characteristic for implementation into forensic practice (Gannon et al., 2004). Considering the potential applied value of pupillary responses as a direct measure of age-specific sexual interest in this context, further research is warranted.

## Footnotes

1 When this analysis was performed *within* category groups, no correlations between pupillary response and appeal ratings were found, all *p*s ≥ 0.06. We attribute this to the low number of images in each stimulus category (five) and the low variance in sexual appeal ratings within categories. For example, male observers’ mean sexual appeal rating for female figures was 5.36 with a standard deviation of only 0.89.
